# When ‘good’ is not good enough: a retrospective Rasch analysis study of the Berg Balance Scale for persons with Multiple Sclerosis

**DOI:** 10.3389/fneur.2023.1171163

**Published:** 2023-06-20

**Authors:** Serena Caselli, Loredana Sabattini, Davide Cattaneo, Johanna Jonsdottir, Giampaolo Brichetto, Stefania Pozzi, Alessandra Lugaresi, Fabio La Porta

**Affiliations:** ^1^Unità Operativa Complessa di Medicina Riabilitativa, Azienda Ospedaliero-Universitaria di Modena, Modena, Italy; ^2^IRCCS Istituto delle Scienze Neurologiche di Bologna, Bologna, Italy; ^3^LaRiCE lab (Gait and Balance Disorders Laboratory), Don Gnocchi Foundation IRCCS, Milan, Italy; ^4^AISM Rehabilitation Center, Italian MS Society, Genoa, Italy; ^5^DATER Riabilitazione Ospedaliera, Azienda USL di Bologna, Bologna, Italy; ^6^Dipartimento di Scienze Biomediche e Neuromotorie, Università di Bologna, Bologna, Italy

**Keywords:** Multiple Sclerosis, postural balance [MeSH], neurological rehabilitation (MeSH), outcome assessment (health care), psychometrics

## Abstract

**Background:**

The Berg Balance Scale (BBS) is one of the most used tools to quantify balance in Persons with Multiple Sclerosis, a population at high risk of falling.

**Aim:**

To evaluate the measurement characteristics of the BBS in Multiple Sclerosis through Rasch analysis.

**Design:**

Retrospective study.

**Setting:**

Outpatients in three Italian Rehabilitation centers.

**Population:**

Eight hundred and fourteen persons with Multiple Sclerosis able to stand independently for more than 3 s.

**Methods:**

The sample (*N* = 1,220) was split into one validating (B1) and three confirmatory subsamples. Following the Rasch analysis performed on B1, the item estimates were exported and anchored to the three confirmatory subsamples. After obtaining the same final solution across all samples, we studied the convergent and discriminant validity of the final BBS-MS using the EDSS, the ABC scale, and the number of falls.

**Results:**

The base analysis on the B1 subsample failed the monotonicity, local independence, and unidimensionality requirements and did not fit the Rasch model. After grouping locally dependent items, the BBS-MS fitted the model (*χ*^2^_8_ = 23.8; *p* = 0.003) and satisfied all requirements for adequate internal construct validity (ICV). However, it was mistargeted to the sample, given the striking prevalence of higher scores (targeting index 1.922) with a distribution-independent Person Separation Index sufficient for individual measurements (0.962). The B1 item estimates were anchored to the confirmatory samples with confirmation of adequate fit (*χ*^2^ = [19.0, 22.8], value of *p*s = [0.015, 0.004]) and satisfaction of all ICV requirements for all subsamples. The final BBS-MS directly correlated with the ABC scale (rho = 0.523) and inversely with EDSS (rho = −0.573). The BBS-MS estimates significantly differed across groups according to the pre-specified hypotheses (between the three EDSS groups, between the ABC cut-offs, distinguishing ‘fallers’ vs. ‘non-fallers’, and between the ‘low’ vs. ‘moderate’ vs. ‘high’ levels of physical functioning; and, finally, between ‘no falls’ vs. ‘one or more falls’).

**Conclusion:**

This study supports the internal construct validity and reliability of the BBS-MS in an Italian multicentre sample of persons with Multiple Sclerosis. However, as the scale is slightly mistargeted to the sample, it represents a candidate tool to assess balance, mainly in more disabled people with an advanced walking disability.

## 1. Introduction

Persons with Multiple Sclerosis (PwMS) are at higher risk of falling than the general population and elderly subjects, with a reported prevalence of falls ranging between 48 and 63% of the assessed population (1). Furthermore, Finlayson et al. found that 63.5 and 82.6% of PwMS reported fear of falling and, respectively, activity curtailment (2). Indeed, studies in the past decades showed that falls in PwMS are consistently associated with impairment of balance (3). The latter is a crucial impairment, which could result in a higher risk of falling and reduced independence in the activities of daily living. To overcome this highly disabling issue, new approaches like virtual reality and exergaming, alongside conventional physiotherapy and rehabilitation interventions, have been widely proposed in several rehabilitative programs and clinical studies, showing significant efficacy in improving balance outcomes (4).

The Berg Balance Scale (BBS) is one of the most used tools to assess balance in PwMS, also within the context of randomized controlled trials (RCT) (5–10). The BBS is a 14-item summative ordinal scale evaluating static sitting balance, postural changes, transfers, and standing balance (both static and dynamic) (11, 12). The classical reliability and validity of the BBS in PwMS were evaluated in two small studies involving 50 subjects and using traditional psychometric procedures. Results showed that BBS had a good concurrent validity with the Dynamic Gait Index (*r* = 0.780) and the Timed Up and Go test (*r* = 0.620) (13). Furthermore, it discriminated between fallers and non-fallers but with a low level of sensitivity (5). The instrument’s reliability was reported as excellent within inter-rater and test–retest reliability analyses (Intraclass Correlation Coefficient = 0.960) (13). On the other hand, Gervasoni et al. in 2016 calculated a minimal clinically important difference cut-off score for the BBS of 3 points. It demonstrated only a moderate accuracy (AUC 0.65) in predicting “responders” (i.e., persons that felt improved after treatment according to a 10% change on the Activity-specific Balance Confidence (ABC) scale submitted pre and post-rehabilitative treatment) versus “non-responders” (persons that felt not improved). This result evidence a suboptimal correlation between BBS balance assessment and persons’ perception of balance improvement (14).

Unfortunately, these traditional psychometric procedures cannot assess and confirm some crucial assumptions and requirements underlying rating scales such as the BBS (15). Indeed, since the sixties, amongst new psychometric methods that have been developed, Rasch analysis has emerged as a powerful tool for assessing the measurement quality of a scale. Mainly, it allows operationalizing the formal axioms of ‘additive conjoint measurement’ using the mathematical model (i.e., the Rasch model) upon which it is based (16). Within the Rasch Measurement Theory (RMT) framework, if a scale displays adequate internal construct validity, the total score will become a sufficient statistic that can be transformed into an interval scale of measurement of ability (17) with a proper unit of measure (i.e., the logit). The scale calibration based on this unit is characteristically independent of the sample distribution employed to calibrate the scale. Interval scales constitute a tremendous advantage as they allow, unlike their ordinal counterparts, both the correct interpretation of change scores and the proper access to parametric statistics, as required in RCT (18). Furthermore, the RMT analytical paradigm (i.e., Rasch analysis) allows assessing in-depth also reliability and targeting so that it is possible to conduct a detailed assessment of the measurement quality of a rating scale.

As the measurement characteristics of the BBS have never been assessed in PwMS within the Rasch model context, the goal of this study is the evaluation of the measurement properties of the BBS in a multicenter sample of PwMS through Rasch analysis.

## 2. Materials and methods

### 2.1. Study design and participants

Data were collected retrospectively within the outpatient Neuro-rehabilitation services of three Italian centers:

- Don Gnocchi Foundation, Milan (DGF);
- IRCSS Istituto delle Scienze Neurologiche, Bologna (ISNB);
- Associazione Italiana Sclerosi Multipla, Liguria (AISM).

Each center screened the digital records of all consecutively admitted patients from 2004 to 2021, including those meeting the following inclusion criteria:

- clinically or radiologically definite relapsing–remitting (RR) or secondary (SP) or primary progressive (PP) Multiple Sclerosis (11);
- ability to stand independently in an upright position for more than 3 seconds;
- at least one complete BBS assessment (i.e., with no missing data) per patient.

The study followed the principles outlined in the Helsinki declaration (12). The Local Ethical Committees of the participating centers approved the conduction of the study (CE-AVEC PG0125189_2022). Written informed consent was sought from the participants according to the Italian Data Protection Authority regulation for retrospective studies (Aut. Gen. n. 9/2016).

### 2.2. Data collected

The BBS is a 14-item summative ordinal scale evaluating sitting balance, postural changes from sitting to standing and vice versa, transfers, and various other standing balance tasks (19, 20). Each item is scored from 0 (cannot perform the task) to 4 (best possible performance) in the observed activity. Thus, the BBS total score ranges from 0 (lowest balance ability) to 56 (highest balance ability). The BBS was administered in each center by licensed physiotherapists. All raters had been adequately trained based on the written scoring guidelines of the BBS to minimize inter-rater variability.

In addition, the rating of the following instruments was also collected:

(1) The Expanded Disability Status Scale (EDSS) score, which quantifies disability in PwMS in terms of the impact of functional systems impairments in determining limitations in activities of daily living, including walking (21);
(2) The Activity-specific Balance Confidence (ABC) scale, which is a structured questionnaire that quantifies the individual’s confidence in performing activities (22);
(3) The number of falls recorded within the 2 months before the BBS assessment.

ABC and fall data were available only from two of the three centers (DGF and ISNB).

### 2.3. Preliminary analyses

#### 2.3.1. Descriptive statistics of the sample

Descriptive statistics for persons’ demographic and clinical variables were performed. In addition, mean ± standard deviation (SD), median with first and third quartile, and absolute frequency with percentage were calculated for the interval, ordinal, and nominal variables, respectively.

#### 2.3.2. Assessment of unidimensionality

##### 2.3.2.1. Classical item analysis

Firstly, we assessed the internal consistency of the pooled sample by calculating the following statistics:

- At the total score level: Cronbach’s alpha (23), where values between 0.70 and 0.95 are considered satisfactory (24);

At the item level:

o The average of inter-item correlations, using Spearman’s correlation coefficient (25), that is the mean of the inter-item correlations between each pair of items. Values ≥0.2 are recommended (26);
o Cronbach’s Alpha if an item is deleted, where values above the total Cronbach’s Alpha are expected to indicate that the item was not internally consistent with the other items (27);
o The item-to-total correlations, based on Spearman’s rho, that are the correlations between each item and its rest score (i.e., the total score minus the item score). Values≥0.40 are considered acceptable (26).

##### 2.3.2.2. Mokken analysis

To obtain initial information on the scalability of BBS items, we performed a Mokken Analysis (MA) on the pooled sample, a scaling procedure for ordinal items based on the Monotone Homogeneity Model (28). It assumes the unidimensionality of the latent trait and the monotonicity and local independence of responses. Furthermore, it can partition a set of items into Mokken scales using an automated item selection procedure (28). We used the following indicators:

- Item scalability coefficient Hj [normed covariance between the item and the rest scores (28)]: values should be ≥0.3 (recommended default value of positive lower bound c);
- Item-pair scalability coefficients Hij (normed covariance between the item scores): values should be positive for items belonging to the same Mokken scale (29);
- Scalability coefficient H: indicates the overall quality of a scale [i.e., the degree to which the test data follow a perfect Guttman pattern (29)].

At the end of the procedure, the analysis shows the number of scales needed for scaling all items. Should the automated algorithm estimate the need for more than one scale to accommodate all the items, we would consider this information in the following analysis steps.

##### 2.3.2.3. Confirmatory factor analysis

We performed a Confirmatory Factor Analysis (CFA) based on polychoric correlations for ordinal data to assess the BBS fit to a unidimensional model. We calculated the following fit statistics:

- Model chi-square (*χ*^2^): an overall indicator of model fit that measures the discrepancy between the covariance matrices of the model and the sample. For a good fit to the model, the *χ*^2^ probability values should not be significant (30);
- Root Mean Square Error of Approximation (RMSEA): values ≤0.06 indicate a ‘good fit’ for a preliminary assessment of dimensionality (31);
- Standardized Root Mean square Residual (SRMR): values ≤0.08 indicate an ‘adequate fit’ (32);
- Comparative Fit Index (CFI) and non-normed Fit Index (Tucker-Lewis Index – TLI): values >0.95 [0, 1] are considered acceptable (30).

We first tested a one-factor model within the CFA. In case of lack of fit for the base analysis, we would allow the correlation of error terms between pairs of items displaying high modification indices (MIs) (30, 33), which are indicators of local dependence (34–36). Should this modified model fail to fit, we would consider this information as evidence of insufficient preliminary unidimensionality in the subsequent analysis steps.

### 2.4. Rasch measurement theory analyses

#### 2.4.1. Sampling strategy

Considering that the available data included a different number of observations per subject (ranging from one to three), we applied the procedure proposed by Mallinson to avoid time dependency (37). In particular, we created several subsamples from the multicenter data pool by randomly selecting only one observation per individual per subsample. We aimed to maintain the size of each subsample between 250 (minimum) and 300 cases (maximum) to avoid type II and I errors, respectively (38). Then, we compared the obtained subsamples according to the main clinical and demographic characteristics and the distribution of the BBS assessments to confirm the randomization effect in getting comparable balanced subsamples.

We performed the Rasch analysis on the validation subsample, chosen as the one with the largest score range of the scale.

#### 2.4.2. Base Rasch analysis

The BBS data of the validation subsample were fitted to the Rasch model (39). The process of testing statistically whether the data fits the Rasch model’s assumptions and requirements is widely known as Rasch analysis, which has been reported in detail elsewhere (15, 39–43). Within this study, the Rasch analysis was based on the partial credit parameterization of the model, which does not place constraints on the item threshold parameters (44).

A full description of the methods used to interpret the Rasch analysis output is summarized in Supplementary Material 1. Briefly, within this study, the following summary statistics were reported:

- *Fitness to the Rasch model*, which relates to the stochastic invariant ordering of the items and persons. An adequate fit to the model was considered achieved if the Standard Deviation (SD) of the item and the person fit residuals (FitRes) were ≤ 1.4 (45), and the summary item-trait interaction chi-square was not significant (i.e., values were above the Bonferroni correction), thus indicating no deviation from the model’s expectations (31, 46). We also assessed the item characteristic curves (ICC), which showed the difference between the observed and the expected responses predicted by the model for each item, based on the probabilistic relationship between person’s ability and item difficulty (15, 39).
- Internal Construct Validity (ICV) requirements:

1. *Unidimensionality*, which requires that all items measure a single underlying construct (47, 48). This requirement was tested with a t-test on each person’s estimates derived from the residuals of each item that loaded, respectively, positively (>0.3) and negatively (<−0.3) on the first component of the residual principal component (37). We considered strict unidimensionality achieved when both the Proportion of Significant Tests (PST) and the lower bound of the Binomial Confidence Interval for proportions (BCI) were below 5%. In contrast, unidimensionality was considered acceptable when only the BCI was <5%. In the case of a subscale structure obtained after the creation of testlets, further indicators (c, r, A) were evaluated (49).
2. *Monotonicity*, which prescribes that the probability of endorsing a response option indicative of higher ability should increase with the increase of the underlying latent trait (balance).
3. *Local independence*, which prescribes that all the variation among responses to an item is accounted for by the person’s ability only and, therefore, for the same value of ability, there is no further systematic relationship among responses. We considered pairs of items to be locally independent if their residual correlation was above a Local Dependency Relative Cut-off (LDRC), calculated by adding 0.2 to the average of residual correlations after removing each item’s association to itself, equal to 1 (50).
4. *Absence of DIF*, which prescribes that an item must also be invariant across relevant subgroups (or person factors), such as gender or age. In this case, different groups of persons with equal levels of the underlying characteristics within a person factor respond in the same manner regardless of their group membership. We tested the presence of DIF with a two-way ANOVA for each item, where scores are compared across each level of the person factor and different ability levels, as summarized by the class intervals. DIF is present when the value of *p*-values of ANOVA are significantly below the Bonferroni correction (51). We tested the following person factors within the DIF analysis: gender, age, disease duration in years, and disease course.

- Targeting and reliability:

1. *Targeting*, which indicates how well the measurement range of the scale matches the distribution of the calibrating sample (15, 43, 52), here expressed as floor and ceiling effects (52) and targeting index (TI) (52). Targeting was considered good and fair for ranges of TI [−1, +1] and [−2, +2], respectively (52).
2. *Separation reliability*, which is the capacity of the scale to separate persons effectively based on their ability level. It was represented by the Person Separation Index (PSI), Cronbach’s Alpha (*α*) (15, 17, 43, 52, 53), the Distribution-Independent Person Separation Index (DI-PSI) (54), and the number of Distinct Levels of Performance Ability (DLPA) (54). PSI or DI-PSI values ≥0.85 and ≥ 0.70 were considered sufficient for individual-level and group-level measurements, respectively (34, 55, 56).

#### 2.4.3. Post-hoc scale modifications

Should the ICV requirements not be met, the scale would be progressively modified to adjust for the violations of the ICV requirements. In particular, we could employ two different analytical approaches to achieve this goal:

1. A conservative approach, where the structure of the scale is unmodified (the total score range is unchanged), but the statistical adjustment performed affects mainly the conversion of the total score into interval-level estimates of ability. Within this approach, the available techniques include item grouping or ‘testlets’ creation (42, 57, 58) and item splitting (40, 43) to account for violations of local independence and the presence of uniform-DIF, respectively.
2. A structure-modifying approach, where the structure of the scale is actively modified, thus affecting the total score range. Within this strategy, the available techniques include item rescoring (15, 43, 59, 60) and item deleting (58). The former is based on collapsing adjacent response categories of the same item to resolve the monotonicity violation. Furthermore, should rescoring be necessary, we would follow published guidelines (59) to maximize statistical indexes and clinical meaning (42, 60) of the rescoring pattern. Finally, item deletion would be performed in case of persisting misfit to the model despite all the above modifications.

Given that the BBS is a widely used scale, we would first aim at using the conservative approach, resorting to applying the structure-modifying strategies only in case of failure of the former to achieve adequate ICV. Thus, fitness to the Rasch model, ICV requirements, reliability, and targeting were all assessed for the original scale (base analysis) and then, after each scale modification, to ascertain whether adequate model fit was achieved. This process was repeated cyclically until no further changes were needed and/or possible.

Should DIF be detected, the influence of the item/testlet splitting on the person estimates would be tested using the procedure presented by Maritz and colleagues (45). After item/testlet splitting, we would anchor the ‘splitted’ solution on the ‘un-splitted’ one, using an item/testlet free from the DIF, and compare the person estimates of the two solutions, calculating an effect size (Cohen’s *d*) of the paired *t*-test of the difference. A Cohen’s *d* < 0.2 would be considered negligible; thus, the DIF would not be adjusted for (45). Otherwise, the ‘splitted’ solution would be chosen as the final (45).

In the case of a final two-testlet solution, conditional total item-trait interaction chi-squares would be calculated because the unconditional ones are known to be unreliable for sample sizes of 200 or more. Compared to this, the conditional fit statistics remain reliable for sample sizes ≤2,000 (61).

#### 2.4.4. Generalization of the results from the validation subsample to the confirmation subsamples

Should a final fitting solution following the above modifications be found on the validation sample, the replication of this solution would be applied to the other confirmation samples. Operationally, we would proceed:

- To export the item difficulty estimates for the final solution of the validation sample;
- To replicate for each confirmatory subsample the final solution obtained for the validation sample and anchor to it the exported item difficulty estimates;
- To verify the fitting of the sample validation final solution on each confirmatory subsample.

A stable BBS-MS validation could be achieved if an adequate fit was confirmed for all subsamples. In this case, its total score could be transformed into interval-level measurements, whose unit is the logit (21, 25, 42). Otherwise, a new iterative phase of analysis on the validation sample could be performed to find a new fitting solution that would be replicated again on the confirmation samples. In case of failure of further attempts, the available validation would be considered not sufficiently stable and worthy of further confirmation in subsequent studies.

### 2.5. External construct validity analyses

Should a final fitting solution of BBS fitting the Rasch model (BBS-MS) be achieved, we would perform the following external construct validity analyses on a single randomly chosen observation for each subject. These analyses would allow us to understand better the clinical implications of the scale’s measurement properties. In particular, we assessed:

- *The convergent validity*, that was tested by examining the correlation of the BBS-MS measurements with the EDSS, which specifically quantifies disability in PwMS, and with the ABC scale total scores (Spearman’s rho). These correlations were expected to be from ‘strong’ to ‘moderate’.
- *The group differences or discriminant validity,* aiming to demonstrate that the BBS-MS could detect differences in groups known to differ in balance quantity. In particular, we hypothesized that if BBS-MS were a measure of balance, its measures should be significantly different across the following groups:

o EDSS 0–3.5 (no walking disability), EDSS 4-5.5 (some walking disability but able to walk without aids), EDSS ≥6 (walking disability requiring aids or unable to walk);
o ‘Fallers’ vs. ‘non-fallers’ according to the ABC scale score cut-off of 40, proposed by Cattaneo et al. (62) in PwMS;
o Low, moderate, and high physical functioning, according to the ABC scale scores of 50 and 80, proposed by Myers et al. (63) in older adults;
o No falls vs. ≥1 fall in the previous 2 months;
o One fall or less vs. ≥2 falls in the previous 2 months.

Finally, we assessed the distribution of the EDSS scores of the whole sample for each DLPA by the mean of a box-plot chart.

Given the continuous nature of the BBS-MS estimates, we would employ a one-way Analysis of Variance (ANOVA) to compare groups upon confirmation of the normality of the BBS-MS distribution. The size of the differences would be estimated using Cohen’s *d* effect size. In case of a non-normal distribution, we would employ the Mann–Whitney U test to compare two groups. Instead, we would use the Kruskal Wallis test followed by the post-hoc pairwise comparison with the Mann–Whitney U test for three or more groups. In both cases, the corresponding r statistic calculated as a non-parametric effect size would be converted into Cohen’s *d* (64).

### 2.6. Statistical notes, software, and sample size issues

Descriptive statistics, internal consistency, and external validity analyses were performed with SPSS software (version 21 for Windows; SPSS Inc., Chicago, IL; 2004). Mokken analysis was run with the R package Mokken (version 2.8.4). The CFA was performed using the Mplus software (version 6.0. Muthen & Muthen, Los Angeles, CA; 1998–2010).^1^ Given 54 score points for the BBS-MS, it was estimated that 540 observations would guarantee a subject-parameter ratio of 10:1, which is the recommended one for factorial analysis (65). Finally, the Rasch analysis was conducted using the RUMM2030 software (version 5.4 for Windows. RUMM Laboratory Pty Ltd., Perth, Australia: 1997–2010),^2^ employing a pairwise maximum likelihood estimation algorithm. A significance value of 0.05 was used throughout and adjusted for the number of tests by Bonferroni correction (66). A sample size of 250 observations would be sufficient to estimate item difficulty, with *α* of 0.01 to<± 0.5 logits, irrespective of the targeting of persons to the items (38).

To facilitate the interpretation of the Rasch analyses, we employed the RUMM logbook™, an *ad hoc* Excel 2007™ application built with Microsoft Visual Basic™ (67). Besides, to facilitate the interpretation of the absolute values of correlation coefficients, a modified version (68) of the cut-off criteria provided by Pallant (64) was adopted: negligible: 0–0.09; weak: 0.10–0.29; moderate: 0.30–0.49; strong: 0.50–0.79; very strong: ≥ 0.80. Finally, we adopted the following criteria for the interpretation of the effect size coefficients (Cohen’s *d*) (69): small: ≥0.2; medium: ≥0.5; large: ≥0.8. We applied the formulas for converting the r and eta-squared effect size generated by non-parametric statistics into Cohen’s *d*, provided by Rosenthal (70) and Cohen (69).

## 3. Results

### 3.1. Descriptive statistics of the sample

All data were collected from a convenience sample of 814 PwMS, collating three samples from Don Gnocchi Foundation (Milano), IRCSS Istituto Scienze Neurologiche Bologna, and Associazione Italiana Sclerosi Multipla (Genova), providing data from 307, 304, and 203 PwMS, respectively. Given that there were multiple observations per patient for the first two centers, we obtained 1,220 BBS assessments in total (568 observations from Milano, 449 from Bologna, and 203 from Genova). The demographic and clinical characteristics of the whole study sample and each center subsample (subjects) are summarized in Table 1. The BBS and ABC scale median distribution of the entire study sample and the center subsamples (observations) are in Table 2.

**Table 1:** Demographic and clinical characteristics of the whole study sample and the center subsamples (subjects).

	Whole sample (*N* = 814)			Milano (*N* = 307)			Bologna (*N* = 304)			AISM (*N* = 203)			Subsample comparison	
	*N*	%	Mean (SD) Median [Range]	*N*	%	Mean (SD) Median [Range]	*N*	%	Mean (SD) Median [Range]	*N*	%	Mean (SD) Median [Range]	Statistics	*p*-value
Gender	814			307			304			203			*x*^2^_2_ = 0.117	n.s
Males	276	33.9	–	106	34.5	–	101	33.2	–	69	34	–		
Females	538	66.1	–	201	65.5	–	203	66.8	–	134	66	–		
Age (years)	799	–	51.5 (11.8)	293	–	48.2, (11.4)	304	–	52.4 (11.8)	202	–	54.6 (11.2)	*x*^2^_2_ = 39.5	0.000*
			51.0 [17.5, 85]			47.0 [17.5, 75]			53.0 [20, 85]			54.5 [24, 78]		
MS disease course	763			262			304	–		197				
Relapsing-Remitting	317	41.5	–	137	52.3	–	101	33.2	–	79	40.1	–	*x*^2^_6_ = 88.9	0.000*
Secondary Progressive	344	45.1	–	99	37.8	–	146	48.0	–	99	50.2	–		
Primary Progressive	102	13.4	–	26	9.9	–	57	18.8	–	19	9.7	–		
Disease duration (years)	712	–	12.5 (9.1)	223	–	14.0 (8.4)	304	–	10,4 (9.3)	185	–	14.2 (8.9)	*x*^2^_2_ = 41.4	0.000*
			11.0 [0, 43]			13.1 [0, 37.1]			8 [0, 43]			13 [1, 43]		
EDSS score	778	–	–	280	–	–	304	–	–	194	–	–	*x*^2^_2_ = 10.8	0.005*
			5.5 [0, 8]			5.5 [2, 8]			6 [0, 7.5]			5.5 [0, 7]		
Falls	316	–	1.1 (2.5)	264	–	1 (2.3)	52	–	1.6 (3.4)	–	–	–	*x*^2^_1_ = 0.312	n.s.
			0 [0, 20]			0 [0, 20]			0 [0, 15]			–		

AISM, Associazione Italiana Sclerosi Multipla; N, sample number; SD, standard deviation; IQR, interquartile range; MS, Multiple Sclerosis; EDSS, Expanded Disability Status Scale; n.s., non-significant.

Nominal variables (gender, MS disease course) were compared between samples using a chi-square test for independence. Ordinal and interval variables (age, disease duration, EDSS score, and falls) were compared across samples with a Kruskal-Wallis test (Mann–Whitney *U* Test for Falls, given only two subsamples).

*Post-hoc comparisons: MS disease course: Cramer’s V 0.234. Age: Milano vs. Bologna *x*^2^ = 85.6, value of *p* = 0.000; Milano vs. AISM *x*^2^ = 125.4, value of *p* = 0.000. Disease duration: Bologna vs. AISM *x*^2^ = 97.8, value of *p* = 0.000; Bologna vs. Milano *x*^2^ = −102.2, value of *p* = 0.000. EDSS score: AISM vs. Milano *x*^2^ = −54.2, value of *p* = 0.009; AISM vs. Bologna *x*^2^ = −64.6, value of *p* = 0.002.

**Table 2:** BBS and ABC scale median distribution of the whole study sample and the center subsamples (observations).

	Whole sample (*N* = 1220)	Milano (*N* = 568)	Bologna (*N* = 449)									AISM (*N* = 203)					Subsample comparison Statistics	*p*-value
		Unique obs	First obs	Second obs	Third obs	Total obs	Unique obs	First obs	Second obs	Third obs	Total obs	Unique obs	First obs	Second obs	Third obs	Total obs		
BBS scale																		
N	1,219	114	192	193	68	567	160	144	144	1	449	203	–	–	–	203	*x*^2^_2_ = 75.3	0.000*
median	45	45	47	49	49	48	45	40	42	28	42	44				44		
[range]	[3, 56]	[12–56]	[14, 56]	[18, 56]	[25, 56]	[12, 56]	[3, 56]	[4, 56]	[5, 56]	[28, 28]	[3, 56]	[7, 47]				[7, 47]		
ABC scale																		
N	502	8	1	84	–	93	80	88	38	–	206	203	–	–	–	203	*x*^2^_2_ = 1.7	n.s.
median	54	49	63	53	–	52.5	59	50	67	–	56	54				54		
[range]	[0, 100]	[19, 71]	[63, 63]	[0, 98]	–	[0, 98]	[2, 100]	[1, 99]	[21, 99]	–	[1, 100]	[8, 96]				[8, 96]		

AISM, Associazione Italiana Sclerosi Multipla; N, sample number; obs, observation; BBS, Berg Balance Scale; ABC scale, Activities-Specific Balance Confidence scale; n.s., non-significant.

Subsamples were compared through a Kruskal-Wallis test.

*Post-hoc comparisons: BBS: AISM vs. Bologna *x*^2^ = −73.1, value of *p* = 0.014; AISM vs. Milano *x*^2^ = −218.4, value of *p* = 0.000; Bologna vs. Milano *x*^2^ = −145.3, value of *p* = 0.000.

Females represented 66.1% of the sample, and the average age was 51 (SD 11.8). In addition, 45.1% of the PwMS suffered from the Secondary Progressive (SP) course of PwMS, and the average disease duration was 12.5 years (SD 9.1). For the EDSS, the median of the available observations (*N* = 778) was 5.5 [0, 8], and the average number of falls (*N* = 316) was 1.1 (SD 2.5). No statistically significant difference was found between the center subsamples regarding gender and the number of falls in the previous 2 months. Concerning the other demographic and clinical characteristics, Milano patients were significantly younger than those from Bologna and Genova. MS disease courses differed significantly between all three centers, as the prevalence of the RR course was higher for Milano. In contrast, the SP course was higher for Bologna and Genova. In addition, patients from Bologna had a disease duration significantly shorter than Milano and Genova, and the latter showed a significantly lower median EDSS score than others (Table 1).

Data quality was excellent, as only one rating amongst the 1,220 available BBS observations had some item missing data. Considering all ratings, the median BBS score was 45 [3, 56]. However, the comparison between the three centers highlighted that BBS data were significantly different between them (BBS median Milano: 48 [12, 56]; Bologna: 42 [3, 56]; Genova: 44 [7, 47]). The median ABC scale value on the available observations (*N* = 502) was 54 [0, 100]. The inter-center comparison of the ABC scale resulted in a non-significant difference.

### 3.2. Assessment of unidimensionality

#### 3.2.1. Classical item analysis

The internal consistency analysis of the BBS showed a satisfactory Cronbach’s Alpha (*α* = 0.918). Similar findings were found for the average inter-item correlations (=0.468 > 0.200). At the item level, Cronbach’s Alphas if an item deleted were below the α, ranging from 0.917 to 0.906, except for BBS03 (sitting unsupported), which showed a higher value of 0.923. Item-to-total correlations were high (mean value: 0.666) for all items, ranging from 0.534 to 0.807, except for BBS03, whose value was 0.223.

#### 3.2.2. Mokken analysis

The automated item selection procedure within the MA showed the scalability of all the items on one single scale, except for BBS03. Besides, all item-pair scalability coefficients Hijs were positive, except for the pairs BBS03-BBS12 (−0.118) and BBS03-BBS14 (−0.258). Furthermore, all the item scale coefficient Hjs were higher than 0.3, as recommended, with the exclusion of BBS03 (0.194). Finally, the scalability coefficient for the entire scale H was equal to 0.593, which qualifies the BBS as a ‘strong scale’.

#### 3.2.3. Confirmatory factor analysis

The baseline CFA on the whole sample failed to support the scale’s unidimensionality (*χ*^2^_77_ = 2366.4; *p* = 0.000; RMSEA = 0.156; SRMR = 0.146; CFI = 0.923; TLI = 0.909). However, forty pairs of items showed large modification indices. After allowing correlation of the errors within the dependent pairs, it was possible to fit a final model indicating sufficient unidimensionality for a Rasch analysis (*χ*^2^_37_ = 91.1; *p* = 0.000; RMSEA = 0.035; SRMR = 0.020; CFI = 0.998; TLI = 0.996).

### 3.3. Rasch measurement theory analyses

#### 3.3.1. Sampling strategy

**Figure 1:**
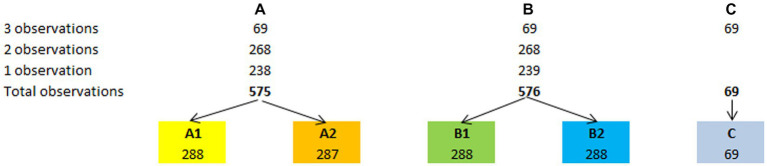
Sampling strategy. The sampling strategy adopted to obtain the validation sample (B1) and the other three confirmation samples (A1, A2, and B2) has allowed a stable validation of the BBS-MS.

Within the 1,220 BBS assessments, 477 patients had only one observation, 268 had two observations, and 69 had up to three observations. As described before, we generated several subsamples across the three centers, each containing only one evaluation per patient (Figure 1), according to the following procedure:

- First, we randomly divided the single observations into two groups (A and B), each containing 238 and 239 observations;
- Then, for each of the 268 patients with two observations, we randomly selected only one of them for sample A, while the left-over observation was segregated into sample B;
- The same procedure was followed for the 69 patients who had three observations. After randomly selecting the first and the second observations for samples A and B, the left-over observation was segregated into a third sample (C; *N* = 69). In this way, we obtained three samples (A–C) of 575, 576, and 69 observations, each containing a single observation per patient;
- Furthermore, to obtain four numerically balanced samples, we randomly divided each A and B sample into two subsamples, thus totaling four subsamples (A1, A2, B1, and B2). All these subsamples included 238 observations, except A2, whose size was 237. The subsample C was then excluded from subsequent analyses because of its insufficient size for performing a Rasch analysis;
- The following subsample comparison to verify the randomization effect showed no statistically significant difference between them (Table 3);
- Since sample B1 had the most extensive score range of the scale (3, 56), it was defined as the validation sample and used for the principal Rasch analysis. On the contrary, samples A1, A2, and B2 were used as confirmation samples to replicate the final fitting solution of B1.

**Table 3:** Comparison of the main clinical and demographic characteristics and the BBS median distribution in the whole study sample and in the randomly generated subsamples.

	Whole sample (*N* = 1,220)	A1 sample (*N* = 288)	A2 sample (*N* = 287)	B1 sample (*N* = 288)	B2 sample (*N* = 288)	Subsample comparisons	
						Statistics	*p*-value
Gender frequencies (%)							
Males	33.5	38.5	32.8	32.6	30.6	*x*^2^_3_ = 0.207	n.s.
Females	66.5	61.5	67.2	67.4	69.4		
Sample size	1,220	288	287	288	288		
Age (years)							
Mean (SD)	51.5 (11.8)	52.1 (11.9)	50.5 (11.9)	52.0 (11.8)	51.6 (11.7)	*x*^2^_3_=3.4	n.s.
Median [range]	51.4 [18, 85]	53 [20, 78]	49.2 [17.5, 85]	52 [26, 85]	51.4 [20, 79]		
Sample size	1,195	283	281	281	284		
MS disease course frequencies (%)							
Relapsing-Remitting	39.1	40.8	38.9	39.5	37.3	*x*^2^_9_=0.459	n.s.
Secondary Progressive	46.5	43.4	48.8	47.4	46.4		
Primary Progressive	14.4	15.8	12.3	13.1	16.3		
Sample size	970	240	244	253	233		
Disease duration (years)							
Mean (SD)	12.6 (9.0)	12.8 (8.9)	12.3 (8.7)	12.2 (9.1)	12.9 (9.4)	*x*^2^_3_=0.8	n.s.
Median [range]	11 [0, 43]	11 [0, 39]	11.5 [0, 38]	11 [0, 43]	11 [0, 43]		
Sample size	1,068	257	245	251	258		
BBS total score							
Median [range]	45 [3, 56]	44 [4, 56]	45 [6, 56]	45 [3, 56]	46 [7, 56]	*x*^2^_3_=0.7	n.s.
Sample size	1,219	288	287	288	288		

BBS, Berg Balance Scale; n.s., non-significant.

Nominal variables (Gender, MS disease course) across subsamples were compared with a chi-square test for independence. Ordinal and interval variables (Berg Balance Scale, Age, Disease duration) between subsamples were compared using a Kruskal-Wallis test.

#### 3.3.2. Rasch analysis

##### 3.3.2.1. Rasch analysis on the validation subsample (B1)

The base analysis performed on the 14 BBS items of subsample B1 (Table 4, subsample B1, Analysis: Base) showed that the scale did not fit the Rasch model (*χ*^2^_56_ = 215.1; *p* = 0.000). The scale failed the unidimensionality requirement, as the percentage of significant t-tests (PST) and the lower bound of the binomial confidence interval for proportions (LBCI) were both >5% (PST = 14.5%; LBCI = 12.6%). Furthermore, one item (BBS13) did not fit the model for under-discrimination (item FitRes: 2.884 > 2.5). Also, the monotonicity requirement was violated because most items had disordered thresholds (12 out of 14, T-DT = 85.7%). Indeed, the BBS scale also failed the requirement of local independence, as there were 19 pairs of items with residual correlations above the local dependency relative cut-off. The only satisfied ICV requirement was the absence of DIF. Finally, the scale was off-target (Targeting Index = 3.288), although its Person Separation reliability Index (PSI = 0.879) was within the cut-off for individual person measurement (≥0.850).

**Table 4:** Summary of Rasch analysis for BBS-MS on sample B1 and replication of the final solution on samples A1, A2, and B2.

Analysis description				Fitness to the Rasch model								Unidimensionality		Targeting and reliability								
				Item fit residual		Person fit residual		Item-trait interaction						Targeting		Separation reliability						
Sample	Analysis name	*N/CI*	*K*	Mean	*SD*	Mean	*SD*	*χ^2^_df_*	*p*	Cut-off ^a^	*p* *Ind-Cond χ^2^*	PST (%)^b^	Lower BCI (%)^b^	SEM^c^	Targeting index^d^	PSI^e^	*α* ^e^	DLPA	DI-PSI^e^	*c* ^f^	*r* ^f^	*A* ^f^
B1 (V)	Base	288/5	14	−0.573	1.628	−0.266	0.893	215.1_56_	0.0000	0.0036	–	14.5	12.6	0.522	3.288	0.879	0.915	7	0.980	–	–	–
B1 (V)	Final	288/5	2	0.312	0.828	−0.244	0.631	23.8_8_	0.0024	0.0250	0.993	3.3	1.5	0.389	1.919	0.768	0.726	5	0.962	0.225	0.951	0.794
A1 (C)	Base	288/5	14	−0.578	1.678	−0.256	0.921	282.8_56_	0.0000	0.0035	–	16.6	14.7	0.524	3.113	0.875	N/A	7	0.980	–	–	–
A1 (C)	Final nAnc	288/5	2	0.489	1.008	−0.276	0.776	18.0_8_	0.0214	0.0250	0.036	2.7	0.8	0.428	1.812	0.762	N/A	5	0.962	N/A	N/A	N/A
A1 (C)	Final Anc	288/5	2	0.173	0.977	−0.312	0.759	19.0_8_	0.0147	0.0250	0.363	1.9	0.0	0.408	1.869	0.751	N/A	5	0.962	N/A	N/A	N/A
A2 (C)	Base	287/5	14	−0.386	1.836	−0.248	0.874	195.1_56_	0.0000	0.0036	–	16.2	14.3	0.500	3.159	0.887	0.919	7	0.980	–	–	–
A2 (C)	Final nAnc	287/5	2	0.243	0.985	−0.236	0.601	18.3_8_	0.0188	0.0250	1.00	2.2	0.4	0.334	1.927	0.730	0.744	5	0.962	0.122	0.985	0.809
A2 (C)	Final Anc	287/5	2	0.620	1.415	−0.260	0.648	22.8_8_	0.0036	0.0250	0.020	4.0	2.2	0.369	1.888	0.769	0.744	5	0.962	N/A	N/A	N/A
B2 (C)	Base	288/5	14	−0.255	1.898	−0.164	0.897	196.6_56_	0.0000	0.0036	–	16.8	14.9	0.524	3.107	0.887	0.926	7	0.980	–	–	–
B2 (C)	Final nAnc	288/5	2	0.207	1.006	−0.243	0.686	18.3_8_	0.0189	0.0250	1.00	1.9	0.0	0.369	1.849	0.748	0.771	5	0.962	0.189	0.809	0.895
B2 (C)	Final Anc	288/5	2	0.381	1.328	−0.282	0.651	22.2_8_	0.0044	0.0250	0.120	3.0	1.1	0.392	1.831	0.774	0.771	5	0.962	N/A	N/A	N/A
Recommended values →					*≤1.4*		*≤1.4*		*n.s.*		*n.s.*	*<5.0* *^b^*	*Lower* *BCI<* *5.0 ^b^*		*[*−*2, +2]*	*≥0.85* ^e^	*≥0.85* ^e^	*≥3*	*≥0.85* ^e^		*≥0.0.70* ^f^	*≥0.70* ^f^

V, validation sample; C, confirmation sample; N/CI, the ratio between sample size and class intervals; K: number of items; SD, standard deviation; *χ*^2^, unconditional chi-square for model fit; df, degrees of freedom; p, Bonferroni-corrected probability value for chi-square; p Ind-Cond *χ*^2^, individual conditional chi-square for model fit; PST, the proportion of significant *t*-test carried out on the estimates that, within a principal component analysis of residuals, loaded positively and negatively (factor loading > 0.30) on the first component; BCI, binomial (95%) confidence interval for proportions of significant *t*-test; SEM, standard error of measurement; PSI, Person Separation Index; *α*, Cronbach’s alpha; DLPA, distinct levels of performance ability; DI-PSI, distribution-independent person separation index (based on DLPA), N/A, Not Available; c, unique variance of the subscales; r, latent correlation between the subscales; A, non-error variance common to the subscales; nAnc, final solution as B1 final, not anchored; Anc, final solution as B1 final, anchored to B1.

In grey summary of Rasch analysis for BBS-MS on the validation sample B1.

Testlet 1, BBS06-BBS07 (standing with eyes closed and with a restricted base of support); Testlet 2, BBS01-BBS04 (postural changes); Testlet 3, BBS12-BBS13 (standing with a restricted base of support and one leg support).

a Bonferroni-corrected value of *p*, which varies by analysis, is used to interpret the corresponding chi-square value of *p*.

b Unidimensionality is achieved when PST is < 5% or when the lower bound of its BCI is < 5%. Unidimensionality is strict when both values are < 5%, whereas it is considered acceptable when only lower BCI < 5%.

c SEM is calculated with the formula: $SD\times \sqrt{1-reliability}$ , where SD is the person’s location standard deviation, and reliability is the PSI with extremes.

d The targeting index is calculated as the ratio between the average person measures and the SEM. Targeting is good, and, respectively, fair, when the average person measure is beyond [−1, +1] and, respectively, [−2, +2] SEM the average item measure (set by default at 0 logits).

e PSI, *α*, and DI-PSI values of ≥0.85 suggest a measurement precision at the individual level. In contrast, a value between 0.70 and 0.849 indicates precision only at the group level (*α* value is not available in case of missing data).

f Values *c*, *r*, and *A* were evaluated to assess the unidimensionality of the subscale structure in the case of the creation of testlets. For *c* (‘unique variance’ for each subscale) low values indicate unidimensionality, whereas for *r* (’ latent correlation’ between the subscales), and *A* (non-error variance common to all subscales) values ≥0.70 indicate unidimensionality.

Considering these findings, we performed the following modifications sequentially, reassessing the internal construct validity assumptions and requirements after each change:

- We did not proceed to rescore items with disordered thresholds to preserve the original structure of the scale, avoiding modification of the original scoring structure of the items and/or deletion of items;
- We accounted for local dependency by creating one testlet for each cluster of items whose residual correlation was above the LDRC. However, as the LDRC decreased by analysis, we created one testlet at a time, proceeding from the item cluster with the highest dependency to that with the lowest. Specifically, in the end, we realized the following testlets:

o BBS01-02-03-04-05-06-08-09-10-11 (postural changes and transfers, sitting position, static and dynamic standing balance);
o BBS07-12-13-14 (standing with a restricted support base, alternate stepping, tandem standing position, and standing on one leg).

After the last modification, the final 14-item scale (BBS-MS) fitted the Rasch model (*χ*^2^_565_ = 485.7, *p* = 0.993; unconditional *χ*^2^_8_ = 23.8, *p* = 0.002; item FitRes SD = 0.828). The scale also satisfied all the ICV requirements regarding monotonicity, local independence, strict unidimensionality (PST = 3.3%; LBCI = 1.5%), and invariance at the subgroup level (no DIF for all person factors assessed: gender, age, disease duration, and disease course).

**Figure 2:**
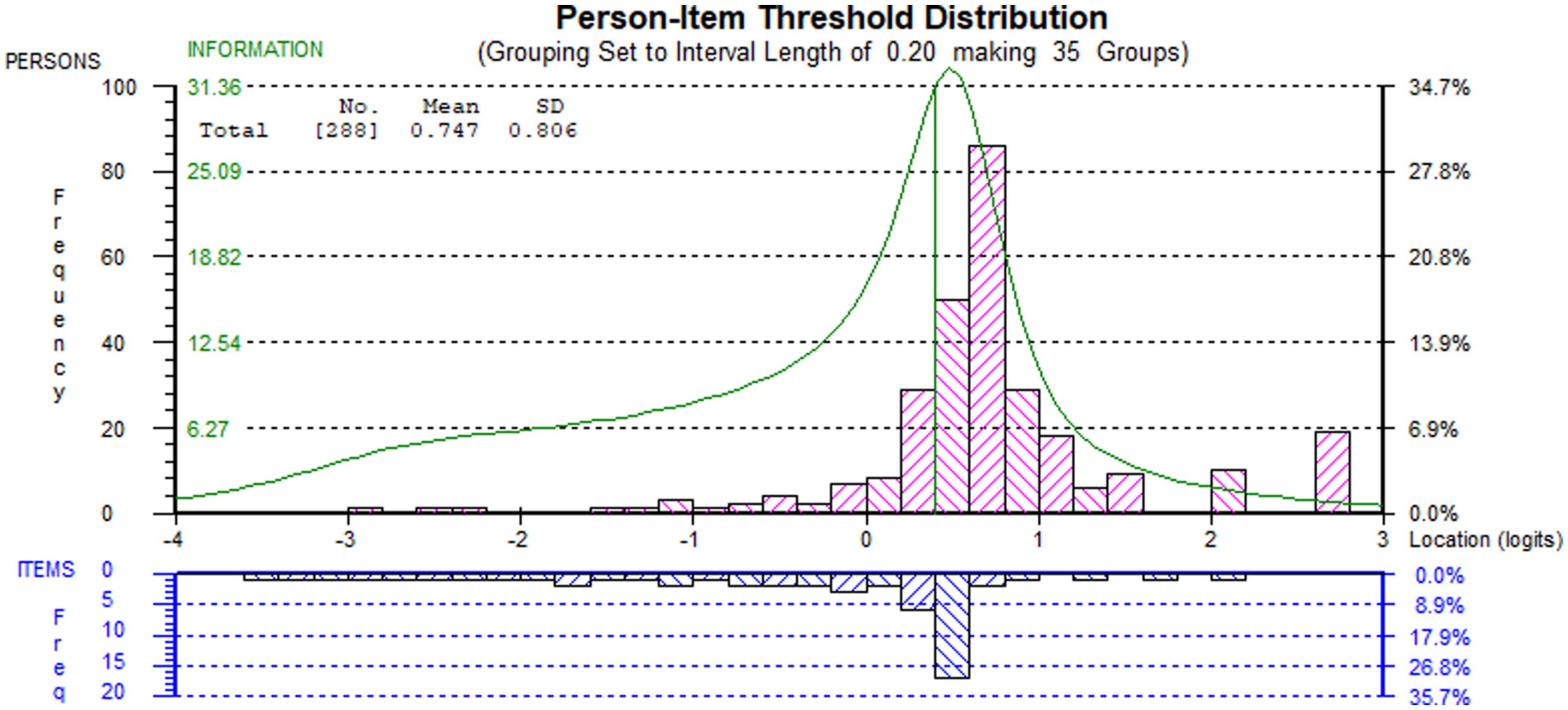
Targeting (person-item threshold distribution) graph of the final solution of BBS-MS on sample B1. Persons (*n* = 288) and item thresholds are displayed, respectively, in the upper and the lower part of the figure, separated by the logit scale.

Although there was no floor effect and a low ceiling effect (7%), the targeting index was 1.922, thus indicating that, on average, the subsample’s ability was above the average difficulty of the BBS-MS items. Indeed, the targeting graph (Figure 2) showed that the persons’ spread, which was about six logits wide ([−2.922, +2.744]), matched fairly the measurement continuum spread, which was about 5.5 logits wide ([−3.437, +2.149]).

The person reliability of the BBS-MS for the validation sample, expressed as Person Separation Index (PSI) and Cronbach’s α, were 0.768 and 0.726. These values were both below the recommended cut-off (≥0.850) for the precision of measurement at the individual level (34, 43, 55). However, as the sample was not normally distributed, we also calculated the number of DLPA using a distribution-free method (54), which considers the measure and the standard error corresponding to each raw score. After controlling for the non-normality of the distribution, the scale could distinguish up to five DLPA. Based on the number of DLPA, we calculated a Distribution-Independent PSI (DI-PSI) of 0.962 (54), which was well above the cut-off for individual person measurement (≥0.850).

##### 3.3.2.2. Rasch analysis on the confirmation samples A1, A2, and B2

To confirm the validity of the BBS-MS, we performed the’ base analysis’ for the confirmation samples A1, A2, and B2 (Table 4). For all three samples, the scale did not fit the Rasch model. It also violated the requirement of unidimensionality, as the percentage of significant t-tests (PST) and the lower bound of the binomial confidence interval for proportions (LBCI) were both >5% for all three samples. At the item level, all but the absence of DIF requirements were violated for all three samples, thus replicating the findings observed for the validating sample. Furthermore, the scale appeared off-target, and the PSI seemed to be slightly below the cut-off for individual person measurement (≥0.850) for all three confirmatory samples (Table 4).

The B1 final solution on each confirmatory sample confirmed the findings observed for the validation sample. After anchoring the exported item difficulty estimates generated from the validating sample to these solutions, the Rasch analyses confirmed the validity of the solution also for the A1, A2, and B2 samples. In particular, the B1 final 14-item scale solution (BBS-MS) fitted the Rasch model as a whole when anchored on each’ base analysis’. It also satisfied all the ICV requirements regarding monotonicity, local independence, acceptable unidimensionality, and invariance at the subgroup level. Although there were no floor effect and a low ceiling effect, the targeting index indicated that, on average, the sample’s ability was above the average difficulty of the BBS-MS items for all three subsamples (Table 4).

The person reliability of the BBS-MS on the three samples expressed both as Person Separation Index (PSI) and Cronbach’s *α* were all below the recommended cut-off (≥0.850) for the precision of measurement at the individual level (34, 43, 55). However, as also these subsamples were not normally distributed, we calculated the number of DLPA using a distribution-free method (54), which yielded five DLPA as for the validation sample B1. Based on the number of DLPA, the DI-PSI was 0.962, which was well above the cut-off individual person measurement (≥0.850) (Table 4).

Finally, we confirmed a stable validation of the BBS in PwMS (BBS-MS) from the above results. The total raw score of the BBS-MS preserved the original BBS range of 0–56. Based on the item calibrations, it was possible to construct a conversion table to transform the BBS-MS raw scores into interval measures of ability (unit of measure ‘logit’) and a 0-to-100 scale, with the associated 95%CI (available in Supplementary Material 2).

### 3.4. External construct validity

#### 3.4.1. Assessment of normality

The assessment of normality conducted on the whole observation sample (*N* = 1,220) confirmed the violation of the normality requirement suggested by the Rasch analysis (Skewness: 0.220; Kurtosis: 2.928; Kolgomorov-Smirnov: 0.175_1220,_ *p* < 0.000; Shapiro–Wilk: 0.877_1220,_ *p* < 0.000). Thus, the subsequent analyses were performed employing non-parametric statistics.

#### 3.4.2. Convergent validity

The analysis of the convergent validity showed that BBS-MS ‘strongly’ correlated directly with the ABC scale (rho = 0.523, *p* < 0.000, *n* = 393) and inversely with the EDSS (rho = −0.573, *p* < 0.000, *n* = 734).

#### 3.4.3. Discriminant validity

A Mann–Whitney *U* test revealed a statistically significant difference in the BBS-MS estimates (0-to-100 scale) across the two groups ‘fallers’ vs. ‘non-fallers’ according to the ABC score cut-off of 40 (*U* = 25125.0, *z* = 7.859, *p* < 0.000), with a large effect size (Cohen’s *d* = 0.873) (Table 5).

**Table 5:** BBS-MS discriminant validity.

	Subgroup comparison				
Independent variable groups*	*N*	BBS-MS median	Statistics	*p*-value	Cohen’s *d*
EDSS 0-3.5	125	71.9	*χ*^2^_2_=204.2	0.000**	1.238
EDSS 4-5.5	221	69.7			
EDSS ≥6	388	65.7			
ABC <40 (fallers)	130	65.7	*U* = 25125; *z* = 7.859	0.000	0.873
ABC ≥40 (non-fallers)	260	69.7			
ABC <50 (low physical functioning)	171	66.1	*χ*^2^_2_=93.0	0.000**	1.111
ABC 50-80 (moderate physical functioning)	149	69.1			
ABC >80 (high physical functioning)	68	72.0			
No falls	208	73	*U* = 6809; z = −4.917	0.000	0.583
Falls ≥1	100	67.9			
Falls ≤1	253	71.9	*U* = 4236; *z* = −4.557	0.000	0.539
Falls ≥2	55	67.3			
DLPA 1	8	7.0	*χ*^2^_4_=286.8	0.000**	1.229
DLPA 2	27	6.5			
DLPA 3	84	6.5			
DLPA 4	445	6.0			
DLPA 5	472	4.5			

BBS, Berg Balance Scale; EDSS, Expanded Disability Status Scale; ABC scale, Activities-Specific Balance Confidence scale, DLPA, Distinct Levels of Performance Ability.

Subgroups were compared through a Kruskal-Wallis test (Mann–Whitney U Test for Falls, given only two subsamples). The number of falls is related to the previous 2 months (not available for the AISM center).

* Dependent variable: BBS-MS estimates, except for DLPA, where the dependent variable was EDSS scores.

** Post-hoc comparisons: EDSS 0–3.5 vs. 4–5.5: value of *p* = 0, Cohen’s *d* = 0.408; EDSS 4–5.5 vs. ≥6: value of *p* = 0, Cohen’s *d* = 0.926; EDSS 0–3.5 vs. ≥6: value of *p* = 0, Cohen’s *d* = 1.317.

Low vs. moderate physical functioning: value of *p* = 0.000, Cohen’s *d* = 0.652; moderate vs. high physical functioning: value of *p* = 0.000, Cohen’s *d* = 0.723; low vs. high physical functioning: value of *p* = 0.000, Cohen’s *d* = 1.540.

LDPA 1 vs. 4: value of *p* = 0.004, Cohen’s *d* = 0.35; LDPA 1 vs. 5: value of *p* = 0.000, Cohen’s *d* = 0.301; LDPA 2 vs. 4: value of *p* = 0.000, Cohen’s *d* = 0.832, LDPA 2 vs. 5: value of *p* = 0.000, Cohen’s *d* = 0.772; LDPA 3 vs. 4: value of *p* = 0.000, Cohen’s *d* = 1.609; LDPA 3 vs. 5: value of *p* = 0.000, Cohen’s *d* = 1.528; LDPA 4 vs. 5: value of *p* = 0.000, Cohen’s *d* = 3.419.

A Kruskal-Wallis test revealed a statistically significant difference in the BBS-MS estimates (0-to-100 scale) across the three levels of physical functioning according to the ABC score cut-off of 50 and 80 (*χ*^2^_2_ = 93.0, *p* < 0.000, Cohen’s *d* = 1.111). In addition, the post-hoc pairwise comparisons of the BBS-MS estimates amongst the three ABC groups were significant, with medium and large effect sizes (Table 5).

A Mann–Whitney U test revealed a statistically significant difference in the BBS-MS estimates (0-to-100 scale) across the two groups’ no falls’ vs.’ ≥ one falls’ in the previous 2 months (*U* = 6809.0, *z* = −4.917, *p* < 0.000), with a medium effect size (Cohen’s *d* = 0.583). A further Mann–Whitney U test revealed a statistically significant difference in the BBS-MS estimates (0-to-100 scale) across the two groups’ 0–1 falls’ vs.’ ≥ two falls’ in the previous 2 months (*U* = 4235.5, *z* = −4.557, *p* < 0.000), with a medium effect size (Cohen’s *d* = 0.539) (Table 5).

A Kruskal-Wallis test revealed a statistically significant difference also in the BBS-MS estimates across the three main EDSS groups (*χ*^2^_2_ = 204.2, *p* < 0.000, Cohen’s *d* = 1.238). The post-hoc pairwise comparisons of the BBS-MS estimates amongst the three EDSS groups were significant, with small and large effect sizes (Table 5). We also observed that when the BBS-MS DLPA was set as the independent variable, there was a statistically significant difference in the EDSS score across the five DLPA, as shown with the Kruskal-Wallis test (*χ*^2^_4_ = 286.8, *p* < 0.000, Cohen’s *d* = 1.229). However, the post-hoc pairwise comparisons of the BBS-MS estimates amongst the five DLPA groups were only partially significant, with medium and large effect sizes (Table 5).

**Figure 3:**
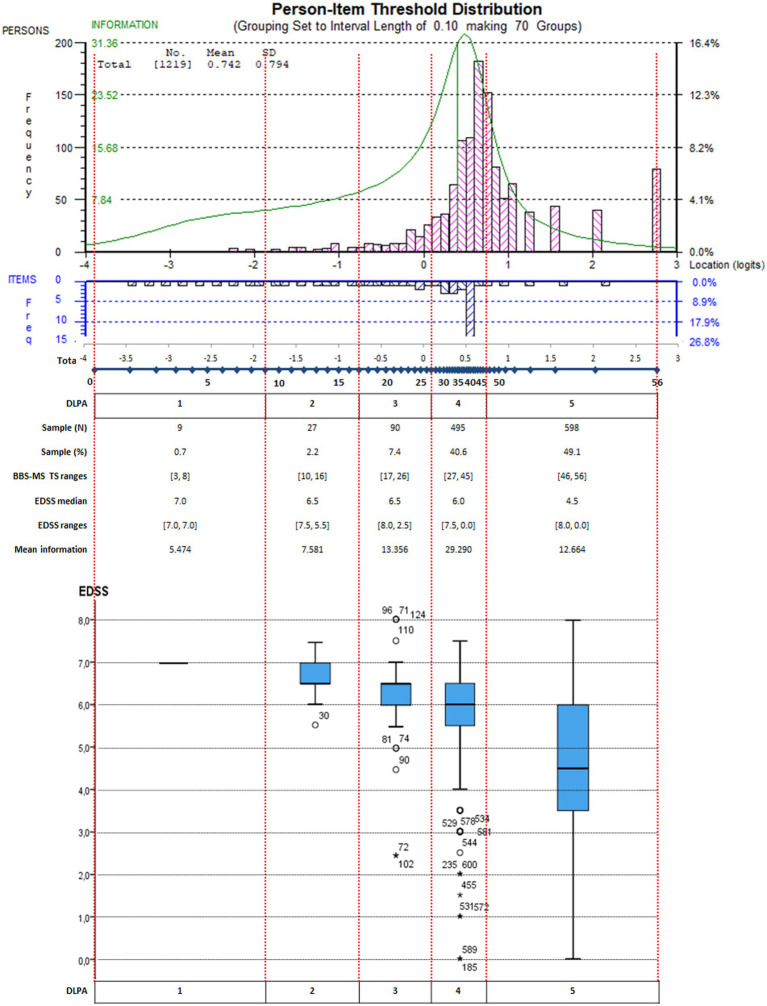
Targeting graph of the BBS-MS integrated with the EDSS score distribution for each DLPA on the whole sample. Persons (*n* = 1,219) and item thresholds are displayed, respectively, in the upper part of the figure, separated by the logit scale. In addition, the distinct levels of performance ability (DLPA) are also indicated. In the lower part of the figure, the EDSS score distribution for each DLPA on the whole sample is represented through a box-plot chart.

In particular, the lower part of Figure 3 shows the actual relationship between the distribution of the EDSS levels across the BBS-MS DLPA for the whole sample (*N* = 1,219). Notably, the EDSS median decreased progressively from the 1st to the 5th as expected, although the EDSS ranges of the 4th and 5th DLPA covered all the scale range with the attribution of high EDSS scores to people with a high level of balance. For example, in the 5th DLPA, 12 persons showed an EDSS score ≥ 7 (walking disability requiring aids or unable to walk) with a BBS-MS TS ≥46. The presence of subjects with extreme scores also caused this unexpected distribution. Besides, in the 4th LDPA area of the measurement continuum, the average test information (i.e., the precision of measurement) was the highest (mean = 29.290; range: [19.237, 32.653]), accommodating about 41% of the sample, whereas the 5th LDPA area included another 50% of the sample with lower mean information (12.664). Despite the good match between the highest information with the peak of the sample distribution, the targeting index was only fair (1.908), as the average ability of the sample was above the average difficulty of the BBS-MS items. Consequently, the graph showed four groups of more than 100 subjects in the LDPAs mentioned above (Figure 3), which the scale could not separate precisely based on their level of balance.

## 4. Discussion

To our knowledge, this is the first study reporting on the Rasch analysis of the BBS for PwMS, as the only previously published assessment was performed by Tesio et al. on some items of the BBS. In particular, they used some BBS items to construct a new tool for measuring balance in PwMS (71). Indeed, in this study, we thoroughly evaluated the internal construct validity, reliability, and targeting of the BBS in a sample of PwMS composed of observations from three Italian centers. Using a validation subsample and three further confirmation subsamples, we demonstrated that, maintaining the original scale item content and scoring structure and after accounting for local dependency, the BBS-MS fitted the Rasch model satisfying all requirements for adequate ICV. On the other hand, the scale was slightly mistargeted to our convenience sample as its items were, on average, less difficult than the mean ability of the PwMS sample. For this reason, it appeared to be reliable for individual person measurement only if we consider the Distribution-Independent PSI, leaving aside the right-skewed distribution of the sample was ignored.

The 814-person sample employed in our analysis was enrolled across the Neuro-rehabilitation services of three Italian centers. It was primarily composed of middle-aged female PwMS, with a higher prevalence of chronic cases with a secondary-progressive course. These epidemiological characteristics are similar to other samples described in the literature (72). Thus, considering the study’s multicenter nature, our sample could be regarded as representative of the PwMS population.

All preliminary analyses (item classical descriptive statistics, Mokken analysis, and CFA) suggested that BBS03 (sitting unsupported) was the item that contributed less to the operational definition of the construct ‘balance in MS’. Indeed, BBS03 had the lowest correlation value with the BBS total score and determined the highest increase in Cronbach Alpha in the case of its deletion. According to Mokken Analysis, BBS03 was the only item not scalable on the same scale as the other thirteen items. It showed negative item-pair scalability coefficients Hijs with BBS12 and BBS14 and an unsatisfactory value of item scale coefficient Hjs (<0.3). Within the CFA, achieving a fitting unidimensional solution for BBS was possible only after allowing the correlation of errors between forty locally dependent item pairs, a quarter of which included BBS03. Local dependence can be induced by multidimensionality between items (43, 73, 74). Indeed, from a clinical point of view, BBS03 is likely to be influenced by construct ‘trunk control in sitting position’, which seems to represent a fundamental prerequisite for ‘balance’ but a separate construct from the latter (31, 42). By the way, this misfit of BBS03 in the internal construct validation of the BBS in neurological patients [three mixed samples in rehabilitation inpatients (31, 42, 75) and Parkinson’s Disease (47)] had already been demonstrated in previous studies.

The sample available for analysis included 1,220 observations for the presence of multiple observations for most patients. We avoided the subsequent time dependency due to repeated assessments from the same patient by randomly generating four subsamples, each including only one observation per patient. This sampling strategy (37) resolved the time dependency issue and allowed us to address several methodological and statistical issues. In particular, it allowed us to obtain a stable calibration of the BBS for PwMS, thanks to the possibility of confirming the final solution of the validation subsample on three further confirmation subsamples. Furthermore, it canceled out inter-center differences in age, disease courses and duration, and BBS total scores.

Regarding the Rasch analysis, the base analysis showed a violation of the monotonicity requirement for most items of the BBS on all four subsamples (B1, A1, A2, B2). However, to preserve the BBS original structure, by avoiding modifying the original scoring structure and/or deleting items, we decided not to rescore items with disordered thresholds and directly address local dependency. This approach was previously followed by Maritz et al., who employed the so-called ‘testlet approach’ in 2019 to revise the internal construct validity of the FIM™ (45). Indeed, such a strategy is particularly advantageous in the case of already published and widespread clinical scales, like the FIM™ or the BBS. In these cases, the preservation of the original structure of the scale is fundamental to guarantee the scale usability by clinicians who are used to the original total score range, the item scoring structure, and the administration of the scale.

We assessed in detail the presence of local dependency in the data, which, despite being a common finding (31), is often not reported and/or not appropriately addressed in Rasch analyses on health outcome scales (58, 73). Recently, the use of an LDRC has been recommended, especially for scales with less than 20 items (as the BBS), since the local dependency may go undetected using the usually recommended absolute cut-offs of >0.3 or even >0.2 (73). Indeed, in most of the previously published reports on the Rasch analyses of the BBS, violations of the local independence requirement were either not reported (76–78) or not found using the frequently recommended absolute cut-off of 0.3 for flagging significant item residual correlations (31). However, in another Rasch analysis of the BBS in Parkinson’s Disease (PD) (47), several pairs of items had residual correlations indicative of local dependency only according to a relative cut-off, like the one employed in this study. It is important to check (and adjust) for local dependence in the data, regardless of the fitness to the Rasch model. This correction is mandatory because it is well known that unadjusted local dependency may bias person estimates, inflate reliability, and negatively affect change scores (73).

The reliability of the BBS-MS for all four subsamples (PSI < 0.80) appeared lower in comparison to those reported for other neurological samples, like a PD sample (PSI = 0.894; strata = 4.6) (47) and a mixed neuro-rehabilitation sample (PSI = 0.952; strata = 7.1) (31). It is essential to highlight that these reliability indices are not an absolute property of the scale but are heavily influenced by the distribution of the calibrating sample (15, 17). Indeed, as the PSI assumes that samples are normally distributed (54), separation reliability will be reduced when items are mistargeted, as for BBS-MS (15). We circumvented this problem by employing a distribution-free method (54), which allowed us to demonstrate that the scale could distinguish up to five statistically DLPA, with a Distribution-Independent PSI of 0.962, which was well above the cut-off individual person measurement (≥0.850).

The BBS-MS was not well-targeted to the sample, given the prevalence of higher-ability PwMS. The tendency towards a ceiling effect for the BBS was already pointed out in several papers reporting on the Rasch analysis of BBS across multiple conditions (47, 62). In particular, it was suggested that this might be caused by the lack of items targeting external postural responses to tripping and slips and dynamic walking balance (47). Indeed, this paper confirms that most PwMS in all four subsamples lie in the right part of the measuring continuum, where a limited number of thresholds is available (Figures 2, 3). In practice, the BBS is not precise enough in the measurement continuum area where high measurement precision would be most needed. In turn, this may also provide an explanatory framework for the reported BBS’ low responsiveness to change in the balance of ambulatory PwMS and with relatively little walking disability (79), as clinical changes within the higher DLPAs may be statistically undetectable. Besides, this paper adds to previous findings the definition of statistical DLPAs for the BBS-MS and their linkage to the disability levels provided by the EDSS. Notably, our results demonstrated quite clearly that the precision of measurement of the BBS-MS within the 4th and 5th DLPA was not optimal in separating persons with a high level of balance.

The lack of items measuring external postural responses and dynamic balance, which are crucial at this high level of balance, is one of the main shortcomings of the BBS. For instance, scales such as the Fullerton Advanced Balance Scale (FABS) (80) and the Unified Balance Scale (UBS) (42) were created to overcome these shortcomings. In particular, the FABS was developed to test both static and dynamic balance under varying sensory conditions in outpatient older adults (80) or affected by other neurological disorders (42, 81). On the other hand, the UBS was devised by pooling items from the BBS, Performance Oriented Mobility Assessment, and FABS, thus creating an activity-based bed-to-community balance scale. The knowledge that the content coverage of the BBS is inadequate for measuring the higher levels of balance required, for instance, during walking and/or in different sensory conditions, has both clinical and research implications. From a clinical point of view, clinicians should consider the administration of additional scales to obtain a proper balance assessment for higher-ability PwMS. Regarding research, it should be assumed that the efficacy of balance rehabilitation for preventing falls in RTCs for PwMS may have been biased unpredictably both by these targeting and responsiveness issues (5–10) and the misuse of parametric statistics applied to BBS ordinal scores (82, 83).

The external analyses confirmed the expected strong correlations of BBS-MS estimates with the EDSS and the ABC total scores. Indeed, EDSS quantifies disability in PwMS considering the alteration of the functional systems, which determine limitations in activities of daily living, including walking. This is not surprising considering that balance is a prerequisite to performing effectively basic activities of daily living, and it is fundamental for adequate stability during gait (72, 84). Indeed, balance requires the integration of several functions controlled by the central nervous system, which can all be affected by MS. In particular, the latter can determine impairments of vestibular function, proprioception, vision, eye movement control, coordination, cognition, and strength. These impairments and/or the disruption of the integration of the underlying functions can frequently lead to balance dysfunction in PwMS (72). The interrelated problems determining this balance dysfunction include a decreased ability to maintain a posture, narrow limits of stability, delayed responses to postural perturbations, and impaired dual-task motor and cognitive integration.

Furthermore, reduced gait speed, impaired dynamic balance, and reduced walking-related physical activity are described as determinants of gait changes (72). These considerations align with the inverse correlation between balance (BBS-MS) and the individual’s confidence in performing activities without losing balance (ABC score), which we found. Indeed, the greater the balance, the greater the individual’s confidence, the lower the fear and the risk of falling, and the better the activity performance (85, 86). At the same time, the lower the balance, the lower the individual’s confidence in performing activities, and the highest the fear and the risk of falling (62, 84, 87–89).

The discriminant validity analysis supports our interpretation of the inter-relationships between impairments, balance, and balance confidence. In particular, BBS-MS estimates are statistically different, with a large effect between EDSS values below 5.5 (absent or partial walking disability) and EDSS values above 6 (severe or complete walking disability) and between the lowest levels of physical functioning (ABC total score < 50), and the highest levels (ABC total score > 70). This result is expected as a balance impairment affects all the gait characteristics (initiation, stability, speed, fluidity, etc.) and, consequently, walking independence in most cases (5, 87).

Regarding the capacity of BBS-MS to discriminate between groups of ‘fallers’ versus ‘non-fallers’, the effect size was only from medium to small. This evidence aligns with previous works, like that by Cattaneo et al. in 2006 (62) in PwMS or by Bogle Thorbahn and Newton in 1996 (90) in older adults. Indeed, they found a poor ability of the scale to categorize subjects into these two groups, showing low sensitivity (0.4 and, respectively, 0.53). The smaller effect size can be explained by considering the risk factor model for falls proposed by the World Health Organization (89), which describes the risk of falling as a multidimensional variable. The model includes interactions between four types of risk factors: biological, behavioral, environmental, and socioeconomic. Balance impairment, measured by the BBS-MS, is a part of the biological domain, which only partially contributes to the fall event’s determination. In summary, a limited effect size of the BBS-MS in the discrimination between ‘fallers’ and ‘non-fallers’ is expected because balance is not the unique determinant of the risk of falling but one of the multiple risk factors interacting with each other.

### 4.1. Study limitation

Although the sample was large and drawn from different populations of PwMS of three Italian Neuro-rehabilitation centers, it should be emphasized that it is a convenience sample. Thus, the possibility of generalizing these findings to other samples may be limited. Another limitation is that the confirmation subsamples were based on different assessments of the same individuals included in the validation sample. Finally, in the external validity analyses, we only used the EDSS score and not the individual functional system subscores, which might highlight different correlations with balance according to the affected functional system.

## 5. Conclusion

This study supports the internal construct validity and reliability of the BBS-MS as a measurement tool in an Italian multicentre sample of PwMS. Using a validation subsample and three further confirmation subsamples, we demonstrated the BBS-MS fitting to the Rasch model and the satisfaction of all requirements for adequate internal construct validity. On the other hand, the scale was slightly mistargeted to our convenience sample as its items were, on average, less difficult than the mean ability of the PwMS sample. In this sense, it also uncovered significant targeting issues that hamper the measurement precision of the scale for PwMS who are still ambulatory and with relatively little walking disability. Indeed, our study suggested that the BBS, even in its Rasch-modified version, may not be a precise and responsive tool for detecting early balance abnormalities in this subgroup of PwMS. However, it is likely to be a precise and responsive tool for PwMS who are more disabled. This information makes the BBS-MS a candidate measurement tool to assess balance in RCTs targeted to more disabled PwMS with an advanced walking disability, together with the availability of interval-level measures of balance ability provided by the Rasch analysis (which allows the use of parametric statistics).

## Data availability statement

The raw data supporting the conclusions of this article are available for download at Zenodo.org (according to the license Creative Commons Attribution 4.0 International) from the following link: https://doi.org/10.5281/zenodo.8029702.

## Ethics statement

The studies involving human participants were reviewed and approved by Comitato Etico di Area Vasta Emilia Centro della Regione Emilia-Romagna (CE-AVEC). The patients/participants provided their written informed consent to participate in this study.

## Author contributions

All authors listed have made a substantial, direct, and intellectual contribution to the work and approved it for publication.

## Funding

The publication of this article was supported by the “Ricerca Corrente” funding from the Italian Ministry of Health.

## Conflict of interest

The authors declare that the research was conducted in the absence of any commercial or financial relationships that could be construed as a potential conflict of interest.

## Publisher’s note

All claims expressed in this article are solely those of the authors and do not necessarily represent those of their affiliated organizations, or those of the publisher, the editors and the reviewers. Any product that may be evaluated in this article, or claim that may be made by its manufacturer, is not guaranteed or endorsed by the publisher.

## Footnotes

^1^ www.statmodel.com

^2^ www.rummlab.com
