# Supplementary material for: When ‘good’ is not good enough: a retrospective Rasch analysis study of the Berg Balance Scale for persons with Multiple Sclerosis
Source: Front Neurol. 2023 Jun 20;14:1171163. doi: 10.3389/fneur.2023.1171163 (PMC10318536; doi:10.3389/fneur.2023.1171163)
Supplement: Supplementary file 1 [file Table_1.DOCX]

Supplementary Material 1 -
Assessment of the measurement quality of an instrument within the Rasch analysis framework

| **Measurement quality domain/parameter** | **Test** | **Expected values/findings** |
| --- | --- | --- |
| **Fitness to the Rasch Model** | |  |
| ***Invariance of the item hierarchy*** |  |  |
| Items: individual item-trait interaction^A^ | Chi-square | Non-significant (Bonferroni corrected) |
| Items: total item-trait interaction^B^ | Chi-square | Non-significant (Bonferroni corrected) |
| Items hierarchy: face validity^C^ | Visual inspection | Item hierarchy conforms to theoretical expectations |
| ***Adherence to the Guttmann Pattern*** |  |  |
| Items: individual ICC^D^ | Visual inspection | Observed probabilities (class intervals) should match expected probabilities |
| Items: individual fit^E^ | Fit Residual | Between -2.5 and +2.5 |
| Items: summary fit residual mean^F^ | Mean of person fit residuals | 0 |
| Items: summary fit residual SD^F^ | SD of person fit residuals | 1 |
| Persons: individual fit^E^ | Fit Residual | Between -2.5 and +2.5 |
| Persons: summary fit residual mean^F^ | Mean of item fit residuals | 0 |
| Persons: summary fit residual SD^F^ | SD of item fit residuals | 1 |
| **Internal Construct Validity requirements** |  |  |
| Monotonicity^G^ | Visual inspection of each item's thresholds | All thresholds are ordered |
| Local independence^H^ | Correlation amongst items' residuals | <Local Dependency Relative Cut-off |
| Unidimensionality^I^ | Paired t-test on PCA of residuals | PST<5% or the lower bound confidence interval (LBCI) PST<5% |
| Absence of Uniform-DIF^J^ | Two-way ANOVA | The main effect is non-significant (Bonferroni corrected) |
| Absence of Non-Uniform-DIF^J^ | Two-way ANOVA | The interaction effect is non-significant (Bonferroni corrected) |
| **Targeting and reliability** |  |  |
| ***Targeting*** |  |  |
| Targeting^K^ | Targeting Index | <|1|: good targeting; >[|1|,|2|]: fair targeting; >|2|: poor targeting (| | indicate absolute values) |
| Ceiling effect^L^ | Calculation of % of persons with maximum score | <2%: good; [2%, 5%]: fair; >5%: poor |
| Floor effect^M^ | Calculation of % of persons with minimum score | 2%: good; [2%, 5%]: fair; >5%: poor |
| Test reliability^N^ | Test information | Expected to follow a normal distribution (higher in the middle of the measurement range) |
| ***Separation reliability*** | |  |
| Distribution-dependent CCT^O^ separation^K^ | Cronbach's alpha | ≥0.70 for group measurement; ≥0.90 for individual person measurement |
| Distribution-dependent separation^P^ | Person Separation Index (PSI) | ≥0.70 for group measurement; ≥0.90 for individual person measurement |
| Measurement error^Q^ | Standard Error of Measurement (SEM) | Expected to be as low as possible |
| Distribution-dependent strata^R^ | Number of strata (H) | ≥2 strata for group measurement; ≥4 strata for person measurement |
| Distribution-independent strata^S^ | Number of DLPA | ≥2 DLPA for group measurement; ≥4 DLPA for person measurement |
| Distribution-independent separation^T^ | Distribution-Independent PSI (DI-PSI) | ≥0.70 for group measurement; ≥0.90 for individual person measurement |

Notes: ^A^Item-trait interaction refers to the Rasch model's statistical property of homogeneity or item invariance, i.e., the items should maintain their stochastic ordering along the whole latent trait[1; 2]. The χ^2^ statistics summarizes whether this important measurement property (invariance) is achieved by comparing the difference between the expected values and observed values across groups representing different ability levels (called class intervals) and across the trait to be measured. When significant (taking into account a Bonferroni-corrected p-value) for an item, the latter violates the invariance of the item hierarchy. This suggests that this item does not fit the Rasch model[3].

^B^This total χ^2^ is calculated by summing up the chi-squares of the individual items (please see note^E^) divided by the sum of their degrees of freedom minus 1[2; 3]. As for the item-trait interaction χ^2^, also for individual items, this χ^2^, when significant (taking into account a Bonferroni-corrected p-value), suggests that the data do not fit the Rasch model as the item hierarchy as a whole does not maintain its stochastic ordering along the whole measurement continuum.

^C^The difficulty order of the items, suggested by the analysis, should make sense from a clinical point of view and be consistent with the expectations derived from theory. If this is the case, it provides evidence of the construct validity of the item set concerning the measured variable[3].

^D^The Item Characteristic Curve (ICC) for a given item displays the expected probabilities to pass the item for any ability level along the measurement continuum. To assess model fit, another curve is constructed by connecting the observed probabilities values across the trait (represented by the various class intervals). The match between the two curves is then assessed[3]. A good match between the two curves suggests normal discrimination. A flatter observed probability curve indicates that the item is under-discriminating, i.e., the responses to the item are too erratic and do not follow the Rasch model's expectations[3]. On the other hand, a steeper curve suggests over-discrimination, i.e., the responses to the item lack the expected randomness and tend to be too deterministic[3]. Independently from item discrimination, individual class intervals markedly outside the expected probability curve suggest that responses to this item, at some ability levels, deviate from the model's expectations. It indicates that the item does not maintain its stochastic ordering along the measurement trait, thus violating the invariance (homogeneity) of the item hierarchy (please see also note^A^)[1; 3].

^E^Fit to the model for individual items and persons is expressed by fit residuals (FitRes), respectively, for items and persons. FitRes are the standardized sum of all differences between observed and expected values summed over all persons for items and overall items for persons[2; 3]. FitRes for a given item (or person) is expected to be 0 if a perfect probabilistic Guttman pattern is achieved. Negative values suggest that the observed responses tend to be deterministic (i.e., lacking the expected randomness). In contrast, positive values indicate that the observed response tends to follow less the expected Guttman pattern (i.e., excessively random). In the case of an adequate model fit, they are expected to be in the range [-2.5, 2.5], representing the 99% confidence interval around the FitRes[3; 4]. Values outside this confidence interval for a given item or person suggest misfit.

^F^Fit to the model for individual items and persons can be summarized as a mean and a standard deviation of the fit residuals, respectively, for items and persons. In case of perfect fit to the model, such means and standard deviations are expected to assume values equal to 0 and, respectively, 1, as they are transformed to approximate a z score, representing a standardized normal distribution[4].

^G^The probability of endorsing an item response indicating higher ability should increase as the underlying level of the latent trait increases (monotonicity requirement)[5]. Consequently, the difficulty thresholds (i.e., transition points between adjacent scoring categories) appear ordered. If the response options for a given item are used inconsistently (e.g., because of misinterpretation of the scoring options, caused by too many scoring options, or inaccurate labeling of the options), the difficulty thresholds appear disordered[2].

^H^All the variation among responses to an item is accounted for by the person's ability; therefore, for the same value of ability, there is no further systematic relationship among responses (local independence requirement)[6]. Items are considered locally dependent if their residual correlation is above a Local Dependency Relative Cutoff (LDRC), calculated by adding 0.2 to the average of residual correlations after removing each item's correlation to itself, equal to 1[7].

^I^All items measure a single underlying construct (unidimensionality requirement)[2; 6]. Unidimensionality is tested post-hoc with a paired t-test on separate estimates for each respondent (derived from subsets of items identified by principal component analysis of the item residuals)[8]. Unidimensionality is considered achieved when the PST (percentage of significant t-test) is <5% (strong unidimensionality) or the LBCI (lower bound of the binomial confidence interval for proportions) is <5% (acceptable unidimensionality)[2].

^J^Item bias or DIF occurs when an item, regardless of maintaining its stochastic ordering at the whole sample level (please see note ^E^), shows a lack of invariance, i.e., DIF, across relevant subgroups (or person factors), such as gender or age[2; 6]. In this case, different groups of persons within a person factor respond differently based on their group membership, despite equal levels of the underlying characteristics. The presence of DIF is tested by a two-way ANOVA for each item, where scores are compared across each level of the person factor and different ability levels, as summarized by the class intervals (please see note ^E^)[4]. In the case of Uniform-DIF (U-DIF), the item bias is systematic along with the trait, as suggested by a significant main effect for the person factor[4]. In the case of Non-Uniform-DIF (NU-DIF), the item bias varies along with the trait, as suggested by a significant interaction effect (person factor × class interval)[4]. Significance p-values are Bonferroni-corrected.

^K^The targeting index is calculated as the ratio between the Person location mean and the SEM (Standard Error of Measurement; please see note^M^ for the formula)[9]. It indicates how the average person location has moved away from the average item difficulty, set by default at 0 logits.

^M^Ceiling and floor effects indicate how many persons in the sample have received the higher and, respectively, the lower score of the scale[9].

^N^Test information (I)[10; 11]for a given person estimate is calculated as follows: $I=\frac{1}{SE^{2}}$ i.e., the reciprocal of the squared standard error around the person measure. It is a measure of how precisely the person ability is estimated[10].

^O^Cronbach's α (Classical Test Theory reliability) is derived as the proportion of variance of the true score and the total variance, including error[3]. It is an indicator of internal consistency because it is a function of the average inter-item correlations[12]. Moreover, this statistic is distribution-dependent[13].

^P^PSI is calculated as the ratio between the variance among the person's estimates of persons and the error variance for each person; it indicates how reliably the persons are separated[3]. Also, this statistic is distribution-dependent, meaning that if the data are skewed for the occurrence of floor and/or ceiling effects, the PSI will be reduced[13]. On the other hand, when the data distribution is not skewed, the PSI and Cronbach's α are virtually identical[7]. ≥0.70 and ≥0.90 are considered the absolute minimum for the group and individual person measurements, respectively[12; 14], as explained in note^N^.

^Q^Closely related to the concept of reliability within the Classical Test Theory framework is that of the Standard Error of Measurement (SEM). That is calculated as follows:$SEM=SD\times\sqrt{1-r}$, where *SD* is the standard deviation of the person measures and *r* is the reliability coefficient (i.e., the Person Reliability Index within the Rasch analysis framework)^[3]^. It indicates the dispersion of the measurement errors when trying to estimate person's abilities from their observed scores. It is less meaningful within the Rasch analysis framework, as in the latter, the standard errors are individually calculated for each person measurement[3]. However, it is here reported as it is used to calculate the targeting index (please see note^Q^)

^R^Strata (H) are the number of statistically distinct levels of person ability (person strata) that the scale can reliably distinguish[15; 16]. This statistic is based on the PSI; therefore, it is distribution-dependent, as it assumes a normally distributed sample[13]. If PSI=0.70, then H=2 (minimum requirement for group measurement); if PSI=0.90, then H=4 (minimum requirement for individual person measurement)[16].

^S^DLPA are the number of statistically distinct levels of person ability that the scale can reliably distinguish independently from the sample distribution [13]. Unlike strata (please see note^N^), this statistic does not assume a normally-distributed sample[13].

^T^DI-PSI is a distribution-independent person separation index calculated using the formula $DLPA^{2}/(1/DLPA^{2})$[13]. This indicator may be useful for skewed samples where the PSI values may grossly underestimate the separation reliability of the scale[13].

**References (Supplementary Material 1)**

[1] D. Andrich, Rasch models for measurement, Sage Publications., London, 1988.

[2] A. Tennant, and P.G. Conaghan, The Rasch measurement model in rheumatology: what is it and why use it? When should it be applied, and what should one look for in a Rasch paper? Arthritis Rheum 57 (2007) 1358-62.

[3] J. Hobart, and S. Cano, Improving the evaluation of therapeutic interventions in multiple sclerosis: the role of new psychometric methods, Health Technology Assessment 2009.

[4] J.F. Pallant, and A. Tennant, An introduction to the Rasch measurement model: an example using the Hospital Anxiety and Depression Scale (HADS). Br J Clin Psychol 46 (2007) 1-18.

[5] M. Mesbah, and S. Kreiner, Rasch models for ordered polytomous categories. in: K.B. Christensen, S. Kreiner, and M. Mesbah, (Eds.), in Rasch Models in Health, ISTE Ltd and John Wiley & Sons, Inc, London UK, Hoboken NJ, 2013.

[6] S. Kreiner, The Rasch model for dichotomous items. in: K.B. Christensen, S. Kreiner, and M. Mesbah, (Eds.), in Rasch Models in Health, ISTE Ltd and John Wiley & Sons, Inc, London UK, Hoboken NJ, 2013.

[7] I. Marais, Local Dependence. in: K.B. Christensen, S. Kreiner, and M. Mesbah, (Eds.), in Rasch Models in Health, ISTE Ltd and John Wiley & Sons, Inc, London UK, Hoboken NJ, 2013.

[8] E. Smith, Detecting and evaluating the impact of multidimensionality using item fit statistics and principal component analysis of residuals. Journal of Applied Measurement 3 (2002) 205–231.

[9] W.P.j. Fisher, Rating Scale Instrument Quality Criteria. Rasch Measurement Transactions 21:1 (2007) 1095.

[10] T. Salzberger, Item Information: When Gaps Can Be Bridged. Rasch Measurement Transactions 17:1 (2003) 910-911.

[11] S. Kreiner, and K.B. Christensen, Person parameter estimation and measurement in Rasch Models. in: K.B. Christensen, S. Kreiner, and M. Mesbah, (Eds.), in Rasch Models in Health, ISTE Ltd and John Wiley & Sons, Inc, London UK, Hoboken NJ, 2013.

[12] J. Brodersen, L.C. Doward, H. Thorsen, and S.P. Mckenna, Writing health-related items for Rasch models - Patient-Reported Outcome Scales for Health Sciences: from medical paternalism to patient autonomy. in: K.B. Christensen, S. Kreiner, and M. Mesbah, (Eds.), in Rasch Models in Health, ISTE Ltd and John Wiley & Sons, Inc, London UK, Hoboken NJ, 2013.

[13] B.D. Wright, Separation, Reliability and Skewed Distributions: Statistically Different Levels of Performance. Rasch Measure Trans 14(4) (2001).

[14] D.A. Revicki, W. Chen, and C.A. Tucker, Developing item banks for patient-reported health outcomes. in: P.S. Reise, and D.A. Revicki, (Eds.), Handbook of Item Response Theory Modeling: applications to typical performance assessments, New York, 2014.

[15] B.D. Wright, and G.N. Masters, Rating Scale Analysis, MESA Press, Chicago, 1982.

[16] W.J. Fisher, Reliability, Separation, Strata Statistics. Rasch Measure Trans 1992 (1992) 238.
