# Supplementary material for: When ‘good’ is not good enough: a retrospective Rasch analysis study of the Berg Balance Scale for persons with Multiple Sclerosis
Source: Front Neurol. 2023 Jun 20;14:1171163. doi: 10.3389/fneur.2023.1171163 (PMC10318536; doi:10.3389/fneur.2023.1171163)
Supplement: Supplementary file 2 [file Table_2.DOCX]

Supplementary Material 2 -

Raw-score-to-measure-estimates conversion table for the BBS-MS

| **BBS-MS raw score** |  | **Logit scale** | **±95% CI** | **0-100 scale** | **±95% CI** |
| --- | --- | --- | --- | --- | --- |
| 0 |  | -3.883 | 1.75 | 0.0 | 13.2 |
| 1 |  | -3.459 | 1.30 | 6.4 | 9.8 |
| 2 |  | -3.149 | 1.07 | 11.1 | 8.1 |
| 3 |  | -2.922 | 0.96 | 14.5 | 7.2 |
| 4 |  | -2.728 | 0.89 | 17.4 | 6.7 |
| 5 |  | -2.547 | 0.86 | 20.2 | 6.5 |
| 6 |  | -2.373 | 0.83 | 22.8 | 6.3 |
| 7 |  | -2.201 | 0.81 | 25.4 | 6.1 |
| 8 |  | -2.033 | 0.80 | 27.9 | 6.0 |
| 9 |  | -1.87 | 0.78 | 30.4 | 5.9 |
| 10 |  | -1.711 | 0.77 | 32.8 | 5.8 |
| 11 |  | -1.559 | 0.75 | 35.1 | 5.7 |
| 12 |  | -1.412 | 0.74 | 37.3 | 5.6 |
| 13 |  | -1.271 | 0.72 | 39.4 | 5.5 |
| 14 |  | -1.136 | 0.71 | 41.4 | 5.3 |
| 15 |  | -1.007 | 0.69 | 43.4 | 5.2 |
| 16 |  | -0.884 | 0.67 | 45.2 | 5.1 |
| 17 |  | -0.767 | 0.65 | 47.0 | 4.9 |
| 18 |  | -0.655 | 0.64 | 48.7 | 4.8 |
| 19 |  | -0.55 | 0.62 | 50.3 | 4.7 |
| 20 |  | -0.45 | 0.60 | 51.8 | 4.5 |
| 21 |  | -0.356 | 0.58 | 53.2 | 4.3 |
| 22 |  | -0.268 | 0.56 | 54.5 | 4.2 |
| 23 |  | -0.185 | 0.54 | 55.8 | 4.0 |
| 24 |  | -0.108 | 0.51 | 56.9 | 3.9 |
| 25 |  | -0.037 | 0.49 | 58.0 | 3.7 |
| 26 |  | 0.028 | 0.47 | 59.0 | 3.5 |
| 27 |  | 0.087 | 0.45 | 59.9 | 3.4 |
| 28 |  | 0.14 | 0.43 | 60.7 | 3.2 |
| 29 |  | 0.187 | 0.41 | 61.4 | 3.1 |
| 30 |  | 0.229 | 0.39 | 62.0 | 3.0 |
| 31 |  | 0.267 | 0.38 | 62.6 | 2.9 |
| 32 |  | 0.302 | 0.37 | 63.1 | 2.8 |
| 33 |  | 0.335 | 0.36 | 63.6 | 2.7 |
| 34 |  | 0.366 | 0.36 | 64.1 | 2.7 |
| 35 |  | 0.393 | 0.35 | 64.5 | 2.6 |
| 36 |  | 0.42 | 0.35 | 64.9 | 2.6 |
| 37 |  | 0.448 | 0.34 | 65.3 | 2.6 |
| 38 |  | 0.475 | 0.34 | 65.7 | 2.6 |
| 39 |  | 0.499 | 0.34 | 66.1 | 2.6 |
| 40 |  | 0.526 | 0.34 | 66.5 | 2.6 |
| 41 |  | 0.551 | 0.35 | 66.9 | 2.6 |
| 42 |  | 0.579 | 0.35 | 67.3 | 2.6 |
| 43 |  | 0.606 | 0.36 | 67.7 | 2.7 |
| 44 |  | 0.637 | 0.36 | 68.2 | 2.7 |
| 45 |  | 0.665 | 0.38 | 68.6 | 2.9 |
| 46 |  | 0.698 | 0.39 | 69.1 | 3.0 |
| 47 |  | 0.735 | 0.41 | 69.7 | 3.1 |
| 48 |  | 0.776 | 0.43 | 70.3 | 3.3 |
| 49 |  | 0.825 | 0.46 | 71.0 | 3.5 |
| 50 |  | 0.883 | 0.51 | 71.9 | 3.8 |
| 51 |  | 0.959 | 0.57 | 73.0 | 4.3 |
| 52 |  | 1.069 | 0.65 | 74.7 | 4.9 |
| 53 |  | 1.25 | 0.81 | 77.4 | 6.1 |
| 54 |  | 1.549 | 1.07 | 81.9 | 8.1 |
| 55 |  | 2.016 | 1.46 | 89.0 | 11.0 |
| 56 |  | 2.747 | 2.07 | 100.0 | 15.6 |

**NOTE.** Abbreviations: 95%CI, 95% confidence interval (equal to 1.96 standard errors of measurement). Person estimates are expressed in logits and 0-to-100 scale. This conversion table can be used only if patients are assessed on all the 14 BBS items.
